# Membrane adsorption in vancomycin treatment is membrane type dependent in CVVHDF: dose correction is crucial

**DOI:** 10.1186/s13054-022-03991-5

**Published:** 2022-04-29

**Authors:** Patrick M. Honore, Sebastien Redant, Pharan Djimafo, Thierry Preseau, Bogdan Vasile Cismas, Keitiane Kaefer, Leonel Barreto Gutierrez, Sami Anane, Rachid Attou, Andrea Gallerani, David De Bels

**Affiliations:** 1 ICU Department, Centre Hospitalier Universitaire Brugmann-Brugmann University Hospital, Place Van Gehuchtenplein, 4, 1020 Brussels, Belgium; 2 ED Department, Centre Hospitalier Universitaire Brugmann, Brussels, Belgium

**Keywords:** Vancomycin, Doses, PAES membrane, AN69ST membrane

Kirwan et al. concluded that target attainment with acceptable trough vancomycin concentrations can be achieved early in treatment with a 2000 mg loading dose and maintenance dose of 750 mg 12 hourly for patients on continuous venovenous haemodiafiltration (CVVHDF) [1]. However, the Kirwan study was completed 7 years ago, using a polyarylethersulfone (PAES) which is well known to be a non-adsorptive membrane [1]. In current practice, more than 95% of treatments with the Prismaflex take place with the adsorptive acrylonitrile 69 surface treated (AN69-ST) [2, 3], which also absorbs significant amounts of vancomycin [2]. Accordingly, using a loading dose of 2000 mg and a maintenance dose of 750 mg/12 h in CVVHDF with the present day standard of practice, using a prismaflex with an AN69ST, could risk treatment failure [2]. In a recent study comparing adsorption of vancomycin in AN69ST versus Polysulphone (PS) (with similar specifications to non-adsorptive membranes such as PAES), there was a significant adsorption of roughly 200 mg in 2 h by AN69ST compared to PS [2]. In vitro, Tian studied vancomycin adsorption of AN69, polyamide, and PS membranes in a hemofiltration model [3]. Vancomycin (36 mg) was added to a volume of a blood-crystalloid (target concentration 50 mg/l) [3]. Adsorption, calculated by the fall in concentration over 120 min, in the 0.6-m^2^ AN69 filters was significantly greater (10.08 ± 2.26 mg) than in the 0.6-m^2^ polyamide (5.20 ± 1.82 mg) or in the 0.7-m^2^ PS (4.80 ± 2.40 mg) filters [3]. Theoretically, an AN69ST membrane could therefore irreversibly take up almost one third of the initial dose [3]. If proven in vivo, loading and maintenance vancomycin doses would have to be adapted accordingly [3]. Because we routinely apply continuous renal replacement therapy (CRRT) using the AN69 ST, we were able to retrospectively demonstrate that daily vancomycin maintenance doses close to 3,000 mg daily were needed during the first 3 treatment days [4]. Choi demonstrated that, within the first hour after changing the membrane, a striking decline in vancomycin trough concentration occurred, which necessitated a considerable increase in the continuous infusion dose [5].

In conclusion, the clinician should be aware of the specific adsorptive properties of the membrane used and dose accordingly. Both personalization of vancomycin dosing and therapeutic drug monitoring for these patients is essential to guide dose scheduling.

## Data Availability

Not applicable.

## Abbreviations

CVVHDF: Continuous venovenous haemodiafiltration
PAES: Polyarylethersulfone
AN69-ST: Acrylonitrile 69 surface treated
PS: Polysulphone
CRRT: Continuous renal replacement therapy
